# Bridging cancer therapies: the role of magnetic nanoparticles in combination cancer therapy

**DOI:** 10.1186/s11671-026-04439-3

**Published:** 2026-02-19

**Authors:** Sajedeh Ebrahim Damavandi, Sayed Mustafa Banihashemi Jozdani, Zahra Elyasigorji, Massoud Vosough

**Affiliations:** 1 Laboratory of Membrane Biophysics and Macromolecules, Institute of Biochemistry and Biophysics, University of Tehran, Tehran, Iran; 2 Department of Regenerative Medicine, Cell Science Research Center, Royan Institute for Stem Cell Biology and Technology, ACECR, Tehran, Iran; 3 Department of Cell and Molecular Biology, Faculty of Life Sciences and Biotechnology, Shahid Beheshti University, Tehran, Iran; 4 Experimental Cancer Medicine, Institution for Laboratory Medicine, Karolinska Institute, Stockholm, Sweden

**Keywords:** Combination cancer treatment, Photothermal therapy, Radiotherapy, Hyperthermia therapy, Photodynamic therapy, Magnetic NPs

## Abstract

Despite the efforts of the medical and research community for effective treatments, cancer is one of the leading causes of death worldwide. Cancer cells’ enduring resistance is a significant cause of treatment failure. One of the most effective approaches proposed to overcome this resistance is a combination therapy. This is a transformative strategy by integrating complementary techniques such as radiation therapy, immunotherapy, photothermal treatment, photodynamic therapy, and hyperthermia, as well as combined with chemotherapy. Numerous studies have investigated the synergistic effects of these therapies to identify the most effective methods for cancer therapy. Researchers also found that magnetic nanoparticles can play a central and innovative role by enhancing the synergistic interactions of combination therapies. Their magnetic reactivity, high surface-to-volume ratio, and surface functionalization enable precise tumor-selective targeting, controlled drug delivery, and efficient conversion of light into heat. They can act as mediators, providing significant benefits when two or more therapeutic methods are used simultaneously. This can enhance their effectiveness. Mechanistically, magnetic nanoparticle-mediated hyperthermia enhances chemotherapy efficacy by elevating tumor temperatures, increasing membrane permeability, and promoting tumor sensitization to radiotherapy. The production of reactive oxygen species (ROS) in cancerous cells, the exacerbation of oxidative damage during photothermal therapy, and the enhancement of immune activation in combined immunotherapeutic approaches improve the effectiveness of chemotherapy. Biocompatible materials such as PEG, chitosan, and dextran can further stabilize these nanoparticles, and ligand functionalization enhances selective tracking of cancer cells. This article provides a comprehensive review of the multifunctional role of magnetic nanoparticles across diverse therapeutic combinations, including radiotherapy, immunotherapy, photothermal therapy, photodynamic therapy, and hyperthermia. It will help those interested in this research topic to comprehensively and validly compare and investigate various studies, make informed decisions, and introduce next-generation Magnetic nanoparticle-based combination therapies for cancer treatment.

## Introduction

The reason for uncontrolled cell proliferation characterizes cancer, which can arise due to genetic mutations, environmental factors, lifestyle choices, and infections [1]. In this regard, recent research has focused on developing new treatment modalities with reduced side effects for these end-stage cancers. Three main alternative therapies were introduced for this aim, which included Hormonal therapies [2] and various kinds of targeted therapies, such as checkpoint-inhibitor therapy [3], photothermal therapy (PTT) [4, 5], hyperthermia therapy [6] and photodynamic therapy (PDT) [4, 7], and radiation therapy [8]. As a nonchemical treatment, Light-based treatments, both photothermal and photodynamic therapies, are designed to selectively kill the cancerous glands in the body through either thermal or oxidative stress, respectively [5].

Notably, most of the therapeutic techniques that were employed in cancer treatment, for example, hyperthermia, represent significant limitations in approaches to functioning effectively on their own. Therefore, its efficacy remains inferior to that of standard treatments and requires specialized equipment. Consequently, specialists offer a combined method, often combined with chemotherapy or radiotherapy, to achieve effective synergistic results and position it as a sensitizing complementary method rather than an independent therapy [6, 9].

Combination therapies have demonstrated improved synergies by combining multiple monotherapies within a single nanoplatform, resulting in highly significant therapeutic effects that are more potent than any single monotherapy or its theoretical combination. To date, various synergistic multimodal therapies have been developed. In particular, combination cancer therapies involving chemotherapy and photothermal, chemotherapy and photodynamic therapy, chemotherapy and radiotherapy, chemotherapy and immunotherapy, and other combination cancer therapies have attracted considerable interest [10].

For example, PTT is a cancer treatment method in which photothermal agents convert near-infrared (NIR) light energy into tumor-killing hyperthermia. The photothermal effect can accelerate the formation of oxygen radicals, thereby enhancing the effectiveness of chemotherapy and providing more effective treatment [11]. However, several issues, such as in vivo uncontrolled heat distribution, tissue or organ heterogeneity, thermal disruption by the circulatory system, and off-target thermal toxicity, can severely limit this performance. Moreover, various properties of thermotolerance and variable temperature sensitivity among different cancer types indicate that even combined therapeutic strategies can be insufficient and need additional complementary solutions [6, 12]. Meanwhile, nanomaterials, such as magnetic nanoparticles, can significantly enhance therapeutic synergy through their unique properties. The unique properties of magnetic nanoparticles make them particularly suitable for combination therapy. They can act as mediators and provide benefits to two or more therapeutic methods used simultaneously, thereby increasing their effectiveness [13].

However, in addition to the several benefits of nanomaterials for enhancing combination cancer therapies, most conventional nanosystems-mediated pathways cannot yet simultaneously provide precise targeted delivery, real-time monitoring, and on-demand external activation, which can be a significant limitation for their therapeutic responses [14–16]. Magnetic nanoparticles (MNPs) have been widely proposed for cancer treatment due to their small size, large surface area, and ability to penetrate tumors when magnetically guided [17]. Coatings such as polyethylene glycol, polyacrylic acid, chitosan, and dextran enhance nanoparticle stability and biocompatibility [18]. Functionalizing charged molecules, hormones, proteins, and high-molecular-weight ligands, such as folic acid, further improves their therapeutic efficacy [19, 20]. By the way, MNPs uniquely combine their magnetic responsiveness, efficient heat generation, and highly versatile surface functionalization within a single platform. These properties can provide remote magnetic guidance, magnetically induced hyperthermia, and image-guided drug delivery, offering spatial precision and multimodal synergy that non-magnetic nanomaterials generally cannot achieve. Recent advances further demonstrate that MNP-mediated multimodal therapy enhances therapeutic efficacy while reducing systemic toxicity, highlighting their superiority as integrated theranostic agents in modern cancer treatment [15, 21–23].

These nanoparticles have focused on improving real-time thermal monitoring and achieving more precise and contactless heat delivery to the tumor. Advances in techniques such as Magnetic Resonance Thermometry (MRT) now make non-invasive 3D temperature monitoring with increasing accuracy *as* a new achievement in cancer treatment, and offer targeted heat sources that reduce side-effects and enhance hyperthermia efficacy [6, 24].

## Physical approaches and chemotherapy in cancer treatments

In the advanced stage of cancer, the tumors are deemed inoperable. Therefore, non-surgical treatment strategies such as radiotherapy, chemotherapy, and photothermal therapy are conventional oncologic plans; however, these are associated with an extreme reduction in patient quality of life [25].

### Radiotherapy

Radiation therapy (RT) is one of the most critical cancer treatment strategies that uses ionizing radiation (IR) to suppress cancer [26]. Due to the complexity of the cancer treatment, approximately 50% of patients with cancer are estimated to receive RT, and it has been reported that the therapy cures 40% of patients [27, 28]. The emergence of radioresistant cancer cells and the RT-induced damage to normal cells threaten cancer therapy and lead to a minor percentage of patients showing a complete response [26, 29].

Over the last decades, significant technological progress in 3D conformal radiation treatments, such as stereotactic (body) radiotherapy (SBRT), intensity-modulated radiation therapy (IMRT), and image-guided radiation therapy (IGRT), has enabled the exact release of matching radiation doses to the accurate places of the tumor while minimizing radiation exposure of surrounding normal tissue [30, 31]. These inventions of various applied technologies of radiography, together with a better acknowledgement of tumor biology at the molecular, cellular, physiological, and immunological levels, have improved the treatment efficacy of radiotherapy, which has led to progress in success from 30% to 80%, particularly in head and neck cancers [30, 32]. However, cancer stem cells are the primary targets of radiation- and chemotherapy-resistance, and tumor heterogeneity plays a significant role in acquired radiation resistance, indicating that radiotherapy still needs improvement [32].

### Photodynamic therapy

Photodynamic therapy (PDT) is a branch of nonchemical cancer therapy that consists of three main components: oxygen, light, and a photosensitizer (PS) [33, 34]. Due to its few side effects, low invasiveness, and increasing rate of the patient’s quality of life [35] PDT has successfully treated multiple types of cancer, including breast, oral tissue, head and neck, skin, esophageal, and bladder cancer [7]. This method can represent a high level of tumor selectivity and the ability to combine PDT with other treatment options, like radiotherapy [4, 36]. Like other cancer therapies, PDT has limitations, including limited light penetration depth, as light can only reach the target area. Due to these limitations, PDT treats cancers that haven’t yet spread to other locations, primarily in skin regions or immediately below organs accessible to a light source.

The choice of light sources, the correct light dose, sufficient PS concentrations, and oxygen play essential roles in PDT [37, 38]. Since the light source must be delivered homogeneously to the affected area to ensure treatment benefits, several laser and non-laser light sources have been tested in PDT [13]. The most frequently used light sources in PDT include laser light sources, which contain three types of laser light sources: argon-pumped, metal vapor-pumped, and solid-state lasers, and non-laser light sources, which include lamp light sources, light-emitting diode (LED), daylight, and X-rays [39].

The PS used in PDT is critical to the treatment outcome. Ideally, an appropriate PS bears tunable amphiphilicity in biological environments. In other words, it should display high molar absorptivity in response to long light wavelengths while being non-toxic in the absence of light irradiation [39]. Silica Nanoparticles (SiO2NPs), liposomes and micelles, carbon nanotubes, peptide-based nanoparticles, bacteriophage nanowires, and graphene are some of the PS PS-delivering nanosystems that improve the capability of photodynamic activity, while overcoming multiple side effects [40].

### Photothermal therapy

Recently, PTT has garnered considerable attention due to its exceptional proficiency in treating cancerous tumors [5]. In PTT, a near-infrared (NIR) laser is used to generate the light energy [41, 42], which leads to electron excitation by the PTT agents. This will result in the receipt of kinetic energy, leading to heat generation in the surrounding PTT medium [5]. Difficult-to-treat tumors as an ultimate target for selective photothermal absorbers can be candidates with minimal invasiveness, in which the induced thermal energy develops cell membrane disruption or protein denaturation of the surrounding tumor cells [42].

The photothermal therapy efficiency is highly dependent on the photothermal transducer, the laser light wavelength, and the mode of laser light delivery. Laser light aims to increase the temperature uniformly in cancerous tissues while preventing damage to healthy surrounding tissues. Photothermal damage to tumor cells typically commences when the temperature reaches 41 °C [43]. However, an effective ablation of the tumors requires reaching higher temperatures (≥ 50 °C) for the destruction of every cancer cell [44, 45]. A significant development in nanotechnology has produced various nanomaterials for nanomedicine and biomedical fields [46]. Some of the nanoparticles, such as gold (Au) and silver (Ag), represent a unique photo-physical phenomenon called localized surface Plasmon resonance (LSPR), which enhances their photo-absorption [47, 48] Therefore, their ability to stimulate hyperthermia in the cancerous cells when exposed to NIR leads them to be considered in PTT [49] Then, the absorbed light energy is converted to heat, leading to damage to tumor cells as well as causing tumor tissue necrosis [50].

### Hyperthermia therapy

Hyperthermia is a cancer therapy technique that uses moderate temperatures of 40–43 °C for approximately 1 h and is considered an excellent, non-specific complementary radiosensitizer and chemosensitizer in multiple tumor types [51, 52]. This method is always combined with either radiotherapy or chemotherapy [53]. Hyperthermia can increase blood flow, improve oxygenation, and enhance the production of oxygen radicals, thereby elevating oxygen levels and altering pH in the cancer, influencing hypoxia, malnourishment, and acidosis. Applying Hyperthermia with or shortly after radiotherapy interferes with the repair of therapy-induced DNA damage, as well as indirectly contributes to tumor cell killing. Furthermore, Hyperthermia activates heat shock proteins (HSPs), triggering protein unfolding, causing loss of functionality and cell death [54]. An appropriate heat delivery system to the tumor site is an important and challenging issue in hyperthermia. The current heat sources include microwave, radiofrequency, laser, and ultrasound. In conventional hyperthermia, a temperature gradient with a maximum at the body’s surface that decreases instantaneously with distance from the external source. Therefore, no thermal discrimination is observed between the targeted tissue and the surrounding normal tissues, which can lead to severe side effects. The development of more effective hyperthermia methods that also reduce these side effects has led to the use of nanoparticles as hyperthermia agents [52].

## Combination therapy and drug resistance in cancer

The emergence of drug resistance restricts the efficacy of antitumor treatments by inducing genetic mutations or changing the tumor microenvironment [55, 56]. In this stage, combination therapy can prevent this resistance by applying multiple methods of treatment at the same time and making cancer cells more susceptible to destruction. This approach could reduce tumor growth and metastasis, elevate the apoptosis rate, and improve overall treatment outcomes [57]. In this article, we provide a comprehensive review of studies using magnetic nanoparticles in combination with cancer treatment modalities, including radiotherapy, photothermal therapy, photodynamic therapy, and hyperthermia.

For example, hyperthermia, as an additional cancer treatment, increases tissue temperature to 40–44 °C. Hyperthermia, in combination with radiation therapy and chemotherapy, allows for a significant reduction in the dose of radiation and chemotherapy required to control a tumor, thereby minimizing damage to normal tissue [58]. Hyperthermia can also represent a practical cytotoxic effect at low pH. When drug carriers are heat-sensitive, hyperthermia stimulates drug release, enabling targeted drug delivery and reducing harm to healthy cells [59]. Also, hyperthermia can raise the blood flow and oxygen supply to the tumor core, increasing drug penetration into deeper tumor areas [9]. However, it is well known that the hyperthermia method may destroy both the tumor and normal cells. Therefore, in combination with nanoparticles (NPs), hyperthermia can overcome the disadvantages of these methods by enabling localization within or around tumor tissue. According to the thermobiological concept, heat can increase the performance of radiation therapy and chemotherapy by increasing drug absorption and making cancer cells more vulnerable to treatment [60].

Several mechanisms explain the synergic effects between hyperthermia and radiation therapy. Hypoxia is one such mechanism in which cells in hypoxic conditions are resistant to radiation but sensitive to heat [61, 62]. Also, cells in mitosis are sensitive to radiation, while cells in the S phase are resistant to radiation but more sensitive to heat [63]. Furthermore, tumor microenvironments are typically nutrient-deficient with an acidic extracellular pH, making them resistant to radiation but vulnerable to heat [64]. When heat is applied externally, normal vascular tissue expands, increasing blood flow, but tumor blood vessels fail to dissipate heat, causing localized hyperthermia at the tumor site [65].

Many clinical studies have explained that incorporating hyperthermia with radiotherapy can significantly improve cancer treatment outcomes. For example, in breast cancer, radiotherapy alone showed an effectiveness rate of 38.1%, and increased to 60.2% with the addition of hyperthermia. Similarly, in primary cervical cancer, the effectiveness of the combined method increased from 58% to 83% [66, 67].

### Magnetic nanoparticles

It is well known that innovative materials, such as magnetic nanoparticles (MNPs), can play a key role in medical science. These nanoparticles can be controlled by magnetic fields and used in hyperthermia, targeted drug delivery systems, and medical imaging devices for diagnosis. In recent years, researchers have also considered these materials essential tools in cancer treatment. The structure of magnetic materials has attracted much attention in various scientific fields, including unique biocompatibility, surface chemistry, non-toxicity, and magnetic moment [68].

MNPs are generally considered non-toxic or low-toxicity compounds due to their chemical composition, physical size, surface chemistry, shape, structure, and biodegradability [69]. MNPs can enter the body through various routes, including the skin, digestive system, bloodstream, and lungs. Intravenous injection is known to be the best way to treat cancer [70]. Several studies have confirmed that MNPs are absorbed more by the liver and spleen than by other organs (such as the brain, heart, kidney, and lungs) after injection [71]. The uptake of charged particles in the liver can be greater than that of neutral particles. Therefore, the charge of MNPs may influence their hepatic clearance. Also, larger MNPs are typically cleared by the liver, spleen, and bone marrow, while smaller ones are removed by the kidneys [72]. In other words, MNPs between 200 and 250 nm in size are typically cleared by the spleen. MNPs smaller than 4 μm are removed by the reticuloendothelial system (RES), primarily in the liver and spleen, and particles larger than 100 nm are phagocytosed by liver cells [73].

### Magnetic nanoparticles as a therapeutic Bridge in combination therapy

MNPs possess unique characteristics that make them a suitable candidate for combined cancer treatment or drug delivery. These features include a large surface area, biocompatibility, and magnetic torque that can be adjusted during manufacturing for defined applications [72]. One reason to consider magnetic nanoparticles in combination therapies is their external stimulation by an induced magnetic field. Due to a broad range of possibilities for combining several instructions in cancer treatment, the intermediation of magnetic nanoparticles for cancer treatment has grown in recent years [74]. On the other hand, magnetic nanoparticles can absorb NIR and convert light into heat [75]. Also, magnetic nanoparticles are used as radiation sensitizers for clinical and therapeutic purposes and in cancer research. Therefore, magnetic nanoparticles improve the effect of radiation sensitivity by enhancing the DNA damage caused by radiation [76]. Table 1 shows some examples of this combination therapy, which will be explained in the following sections.

**Table 1:** The efficacy of combination cancer therapy in different cancer treatments

Cancer Type	Treatment Modality	Combination With	Size / Hydro Dynamic diameter	Surface Coating	Model type	Efficacy Reported (%)	Ref
Prostate and Breast Cancer	Hyperthermia Therapy	Superparamagnetic iron nanoparticles	Not mentioned Almost 100 nm	Fe/C composite	In vitro	50	[77]
Breast Cancer	Photodynamic Therapy	IR780**/**CaO2 Nanoplatform	94.5 ± 2.1 nm	SLN lipid shell	In vitro	73.5	[78]
Liver Cancer	Hyperthermia Therapy	Iron oxide (Fe3O4)	< 20 nm (HD Not mentioned)	Small-molecule coatings (best: 34DABA)	In vitro	61–88	[79]
Head and Neck Cancer	Photodynamic Therapy + Radiotherapy	Immune checkpoint inhibitor (ICI) nivolumab	Unknown	Unknown	Clinical Trial	Case report treatment	[80]
Melanoma	Photothermal Therapy	Iron oxide-gold core-shell	~ 100–150 nm (Not mentioned precisely)	PEGylated liposome	In vitro + In vivo	85	[81]
Glioblastoma	Radiotherapy	Superparamagnetic iron nanoparticles	118 nm	PEGylated shell (no ligand)	In vitro + In vivo	75	[82]
Lung Cancer	Radiotherapy + Hyperthermia	superparamagnetic iron oxide	HD Not mentioned (MMAD Almost 370 nm)	Pluronic F127-CTP + EGFR peptide	In vitro + In vivo	70	[83]
Breast Cancer	Photothermal Therapy	Gold-coated MNPs	About 5 nm	GSH + Pt(IV) + CRGDK	In vitro	80	[84]

#### Magnetic nanoparticles and photothermal therapy

The combination of chemotherapy and photothermal therapy has recently attracted attention [85]. A recent study investigated the effects of magnetic nanoparticles and photothermal therapy on U-87 MG human glioblastoma cells. This study applied temozolomide (TMZ) extracts derived from indocyanine green (ICG) to nanoparticles. The results showed that the developed nanoparticles induced apoptosis in cancer cells using a combined chemotherapy and photothermal therapy approach, thereby increasing the expression of genes associated with apoptosis in brain cancer cells [86]. Some studies showed that NIR irradiation-induced heating can improve drug diffusion. It can sensitize cancer cells to chemotherapeutic drugs, thereby enhancing anticancer effects [87, 88]. A photothermal agent must have specific characteristics, including the lowest toxicity level and the highest biocompatibility. Also, its diameter must be between 30 and 200 nm to remain in the bloodstream for a long time. Also, it must be able to absorb NIR. So, the agent must have a high absorption cross-section to maximize light conversion into heat. Many compounds are photothermal agents, but metal nanostructures, called plasmonics, have unique photophysics. When particles of plasmonic material interact with electromagnetic radiation, the radiation’s oscillating magnetic field causes the conduction band’s electrons to oscillate around the particle’s surface. This oscillation means the release of heat. The amplitude of the oscillations reaches a maximum at a particular wavelength, known as the localized surface plasmon resonance (LSPR). Many magnetic particles can convert light into heat. However, iron oxide nanoparticles are confirmed as suitable for PTT [89]. Iron oxides are chemical compounds such as magnetite (Fe₃O₄) and maghemite (Fe₂O₃), and at specific sizes (25 nm for magnetite and 30 nm for maghemite), both exhibit superparamagnetic behavior. Superparamagnetic nanoparticles are magnetized in the presence of an external magnet, but return to a non-magnetic state when the external magnet is removed [90].

Many studies on magnetic nanoparticles for photothermal therapy have used the first biological window in the near-infrared range. The structure of magnetite, an inverted spinel, shows that the Fe (II) and Fe (III) ions in the octahedral sites of the crystal can cause a gap charge transfer, which leads to the appearance of a second region near the IR range at 1000–1350 nm [90]. Iron oxide nanoparticles are used in the photothermal therapy method in two ways: either they act as PA or are combined with PA hybrid nanoparticles and exhibit near-IR absorption characteristics [91].

Many studies on magnetic nanoparticles for photothermal therapy have used the first biological window in the near-infrared range. The structure of magnetite, an inverted spinel, shows that Fe (II) and Fe (III) ions in the octahedral sites of the crystal can cause a gap charge-transfer, leading to the appearance of a second region in the IR range at 1000–1350 nm. In this window, the radiation is lost to a lesser extent due to radiation scattering and can thus penetrate deeper regions [92]. The studies of Tsai et al. (2018) on magnetite cluster nanoparticles (ION-CNPs) showed weak absorption in the 750–900 nm range and, as already mentioned, observed a more significant charge transfer across the band gap in the second NIR region. In these particles, the photothermal conversion efficiency was 20.8%. Using a 1064 nm laser at 375 ppm, iron resulted in a 33-degree increase in water temperature from 25 to 58 °C [93].

Furthermore, Lu et al. (2018) developed a magnetic nanoparticle-modified porous scaffold containing magnetic SrFe12O19-modified bioglass/chitosan (MBCS) that combines magnetic stimulation with photothermal therapy. Furthermore, this magnetic component enhanced osteogenic gene expression and subsequently supported bone regeneration. At the same time, near-infrared (NIR)–induced photothermal heating from the nanoparticles effectively triggered apoptosis and destruction of osteosarcoma cells. Overall, the MBCS demonstrated significantly improved antitumor activity compared to the single scaffold [94].

#### Magnetic nanoparticles and hyperthermia

Magnetic nanoparticles produce heat by using an external alternating magnetic field (AMF). Therefore, they are used as intermediate compounds in magnetic hyperthermia [72]. At high AMF frequencies, the heat generated by ions is sufficient to reach temperatures above 42 °C. Stable temperature changes above 42.8 °C cause necrosis, destroying many structural and functional proteins [95]. Another application of magnetic nanoparticles is magnetic hyperthermia as a drug-delivery vehicle. When drugs are embedded in a heat-sensitive coating, drug release occurs when the drugs are heated due to hyperthermia [96, 97].

In MNPs exposed to an alternating magnetic field, magnetic energy converts to thermal energy. Heat generation occurs due to hysteresis losses, representing the energy lost during the magnetic cycle [93, 98]. Ferromagnetism is formed by magnetic domains in which all atomic moments are parallel to each other to achieve a lower energy state. While ferromagnets are formed by magnetic domains with opposite magnetic moments in different areas, when an external magnetic field is applied, the magnetic domains tend to align with it. When the applied field is removed, the magnetization does not return to zero; it remains remanent. To reduce the magnetic field strength to zero, a magnetic field of precise strength must be applied [98, 99].

Thermal sensitization simultaneously amplifies the effects of chemotherapy by applying hyperthermia. This enhancement is attributed to several mechanisms, including improved accumulation of anticancer drugs within tumors, which is related to the physiological effects of mild hyperthermia on tumor vasculature. This results in increased blood flow, facilitating the delivery of medication. Although the specific mechanisms underlying enhanced drug cytotoxicity are not yet fully understood, it is generally observed that greater intracellular drug absorption is associated with increased cellular activity. Membrane permeability and inhibition of DNA repair. Studies have shown that increasing temperature maximizes the cytotoxic activity of many anticancer agents in mild hyperthermia [95, 100].

Kawai et al. (2005) reported that magnetic cationic liposomal structures (MCLs) treated glioma cells after heating to 45 °C. Treated cancer cells disappeared completely in glioma-bearing rats when exposed to an alternating magnetic field at 118 kHz and 384 Oe. This experiment was repeated three times, each for 30 min, at 24-hour intervals. They also observed that cancerous cells in areas where MCLs were not injected disappeared completely, due to the generation of antitumor immunity by this method [101].

Niemirowicz et al. (2015) employed a method to increase the anticancer activity of MNPs by attaching Cathelicidin (CLL-37) to their surfaces. They treated DLD-1 and HT-29 cell lines, both colon cancer cell lines, with MNPs. The cell lines treated with CLL-37 have shown a decrease in cell viability compared with CLL-37-free MNPs [102].

#### Magnetic nanoparticles and radiotherapy

Radiation therapy has attracted great interest in cancer treatment. Radiotherapy delivers ionizing radiation at high intensity and with high precision to the tumor tissue, resulting in the death of the tumor cells [103]. However, radiation therapy has disadvantages, including the possibility of injuring surrounding normal tissue. Additionally, some tumor cells far from the radiation site may receive lower radiation intensity, leading to the development of radiation resistance. Mitotically active tumor cells are typically more sensitive to radiation than surrounding healthy tissues. Thus, the minimum dose of radiation needed to kill tumor tissue may only injure but not destroy normal tissue [104].

However, cancer cells’ “radiation resistance” is a significant challenge in radiotherapy failure. Tumor cells do not share a common characteristic of radiation resistance, and radiation can also damage healthy cells during tumor treatment [105]. Promising strategies to overcome these challenges include radiation-protecting compounds for healthy cells and radiation-sensitizing compounds for cancer cells. Many compounds are used as radiation sensitizers in clinical cancer research. Two radiosensitizing strategies are considered. First, the radiation sensitizer increases radiation sensitivity by enhancing the DNA damage induced by radiation. The second strategy involves targeting the DNA damage response signaling pathway or affecting proteins responsible for DNA integrity [106].

Wu et al. (2019) found that a magnetic nanoparticle-based platform modified with a cationic polymer induced cytotoxicity in glioma cells treated with radiation therapy. The magnetic properties of the nanoparticles ascertained that myeloid-derived suppressor cells (MDSC) could take up nanoparticles in the brain tumor. This observation may be due to the destruction of glioma cells, as well as to MDSCs repolarizing from an immunosuppressive to a pro-inflammatory phenotype, which accelerates antitumor effects and synergistically enhances radiotherapeutic effects. This platform can serve as a robust dual-targeting system for glioma radiotherapy by simultaneously eradicating tumor cells and modulating myeloid phenotypes in the central nervous system [107].

#### Magnetic nanoparticles and photodynamic therapy

PDT efficiency depends on forming reactive oxygen species (ROS), which can diffuse through the biological membranes [108]. These interactions induce cell death via apoptosis, necrosis, and other cell membrane damage [109]. ROS can affect nucleic acids, lipids, amino acids, and proteins in tumor tissue and prevent angiogenesis inside solid tumors [110]. In PDT, light is a tool to induce and modulate the selective destruction of malignant tissues or organs. Combining nanomaterials aligned with photosensitizers can overcome these issues with a higher efficiency [108]. Magnetic nanoparticles, such as magnetite (Fe3O4), exhibit several distinct properties that make them suitable nanocarriers for biomedical applications. It is well-known that iron oxide magnetic nanoparticles have been successfully employed as drug carriers for photosensitizers and chemotherapy. These properties include the ability to reach the desired site of treatment using an external magnetic field (magnetic targeting), high surface area, small volume, biocompatibility, chemical stability, and relatively low-cost production [111, 112]. Therefore, the integration of nanoparticles as a carrier of photosensitizers is a promising approach in the PDT method [108].

Hou et al. (2019) developed a multifunctional nanoplatform to enhance the PDT effect by increasing oxygen concentration in tumor cells via the Fenton reaction and targeting the mitochondrial site to reduce the distance between the ROS and the target site.

Fe3O4@Dex-TPP nanoparticles coprecipitate in the presence of triphenylphosphine (TPP)-grafted dextran (Dex-TPP) and Fe^2+^/Fe^3+^. After that, the photosensitizers of protoporphyrin IX (PpIX) and glutathione-responsive mPEG-ss-COOH are grafted on Fe3O4@Dex-TPP to form Fe3O4@Dex/TPP/PpIX/ss-mPEG nanoparticles. In continuation, part of Fe_3_O_4_ is decomposed into Fe^2+^/Fe^3+^ inside the acidic lysosome, and after that, Fe^2+^/Fe^3+^ diffuses into the cytoplasm, where Fe^2+^ reacts with the overproduced H_2_O_2_ to produce O_2_ and •OH. Subsequently, undecomposed nanoparticles enter the cytoplasm via photoinduced internalization and are directed to the mitochondria, where they directly generate ROS. Simultaneously, the O_2_ produced by the Fenton reaction can serve as an input for PDT. Finally, it was observed that Fenton reaction-assisted PDT can significantly improve the therapeutic efficacy against tumor cells [113].

### Radiosensitizers function of ferromagnetic iron oxide nanoparticles

Ferromagnetic iron oxide nanoparticles are called manganite Fe_3_O₄ or maghemite (Fe_2_O₃). Due to their ferromagnetic properties, these nanoparticles have unique properties and interesting capabilities. These nanoparticles can be directed to the desired location using magnetic force. These nanoparticles have high biocompatibility and low tissue toxicity. These nanoparticles can damage DNA and other cellular structures by producing ROS. By catalyzing the production of ROS under ionizing radiation, these nanoparticles enhance RF-induced DNA damage and lead to more significant oxidative stress [114]. Chitosan-ferromagnetic iron oxide nanoparticles carrying the human adenovirus (E1A) gene enhance radiation sensitization of the cervix.

This gene decreases HER-2 expression and increases p53 expression, which is essential in regulating radiation resistance in cancer [115]. These compounds enhance cervical radiation sensitization in xenografted mice. They increase oxidative stress due to ferromagnetic iron oxide nanoparticles [116].

Also, Shetake et al. (2019) presented functionalized oleic acid nanoparticle-sensitized mouse cancer cells (WEHI-164) and human fibrosarcoma cancer cells. These cells were exposed to gamma radiation (2 Gy). The results showed a significant decrease in cancer cell proliferation. The sensitization mechanism involves binding the MN-OA nanoparticle to HSP90, which reduces the expression of proteins encoded by this gene that play roles in cell cycle progression (cyclin B1 and CDC2) and the repair of double-strand DNA breaks. DNA damage is even more stable in cells treated with oleic acid nanoparticles and irradiation with the focal marker γ-H2AX. The efficacy and mechanism of radiosensitisation by these nanoparticles were studied in a mouse fibrosarcoma model. The results showed that HSP90 plays a vital role in the mechanism of radiation sensitization of MN-OA nanoparticles [106]. Khoei et al. (2014) studied two combinations of magnetic nanoparticles with different structural features (dextran and dextran-coated amino groups). This study used the human prostate cancer cell line DU145 with iron oxide nanoparticles at a 1 mg/ml concentration and different doses of 6 MV. The results showed that the uptake of amino group-coated nanoparticles by DU145 cells was significantly higher than that of single nanoparticles. In addition, the cell lifespan decreased with increasing iron oxide concentration. The dose-escalation factor was approximately 1.2 across doses from 2 to 8 Gy for 6 MV X-rays [117]. Therefore, by introducing appropriate surface modifications, iron oxide nanoparticles can further penetrate cancer cells and increase their radiation sensitivity [106]. In another study, Sood et al. used a gold coating of iron oxide nanoparticles. This system combines the magnetic properties of magnetic nanoparticles with surface plasmon resonance properties. The nanoparticles are coated with thiolated pectin (TPGIN). This coating ensures stable nanoparticle release and allows loading of hydrophobic anticancer drugs. This study used gamma rays with a dose of 0.5 Gy and the cervical cancer cell line HeLa. The results showed that TPGIN use significantly increases reactive oxygen species (ROS) production in HeLa cells. The mechanism underlying this phenomenon is increased sensitivity to radiation (0.5 Gy) [118].

One challenge of using radiation sensitizers in clinical settings is timing. So, daily radiotherapy should be performed with the highest drug concentration in the target tissue. To address this issue, Menon et al. developed a novel nanoparticle (NP) containing polylactic co-glycolic acid attached to R11, which can penetrate prostate cancer cells and act as a potent radiosensitizer, 8-dibenzothiophen-4-yl-2-morpholine. Encapsulated 4-yl-chromen-4-one (NU7441). This system can specifically target prostate cancer. Nanoparticle characterization showed that the conjugated NPs (NP R11-NU7441) have an average size of approximately 274,680 nm. The results indicate that R11-conjugated PLGA NPs have the potential to be used as novel platforms for targeted radiosensitisation of prostate cancer cells [119]. Shestovskaya et al. (2024) studied the effects of 2 and 3 Gy rays on HEK293 and HCT116 cell lines, as well as BALB/C mice with an intensity of 5 Gy. In this experiment, the researchers used spherical heparinized iron nanoparticles measuring 20 nm. In this study, the combined effect of radiation and heparinized iron nanoparticles on the cell surface increased the number of cancer cells in the G2/M phase of the cell cycle. This causes problems during mitosis and slows cell division. This number is much higher than the cells that received only a specific dose of radiation or particles. On the other hand, studies of magnetically controlled nanoparticles in BALB/C mice with a CT26 transplant show effective results, demonstrating that intravenous administration of heparinized iron nanoparticles and radiation reduces tumor growth. Nanoparticles with 20.8% T/C, not guided by a magnet, showed a tumor growth rate of 57.9% T/C [120].

## Magnetic nanoparticles in synergic mechanism of hyperthermia and radiotherapy

In magnetic hyperthermia, MNPs can penetrate tumor tissue and be heated by an external alternating magnetic field (AMF) [70]. Through magnetic targeting, MNPs accumulate at the tumor site, making this method suitable for deep-seated tumors [121]. MNPs also offer advantages such as controlled localization, real-time imaging for tracking, and functionalization with ligands for targeted therapy [122, 123].

Hyperthermia combined with MNPs and radiotherapy presents a novel approach to overcoming challenges in cancer treatment. Rezaei et al. (2018) studied the combined effect of hyperthermia (43 °C, one hour) and radiotherapy (2 Gy, 6 MV) on the U87MG glioblastoma cell line using PCL-PEG-coated MNPs loaded with IUdR. The results showed significant inhibition of cancer cell growth. PCL-PEG-coated MNPs acted as a suitable carrier for IUdR and functioned as heat and radiation sensitizers [124].

Attaluri et al. (2015) investigated hyperthermia combined with MNPs and radiotherapy in prostate cancer. Their study of human prostate cancer cell lines (LAPC-4 and PC3) demonstrated that radiation (5 Gy) combined with hyperthermia (43 °C, 1 h) significantly reduced cell survival compared to either treatment alone. Notably, PC3 cells were less sensitive to the combination treatment than LAPC-4 cells, which exhibited approximately 100 times lower survival under identical conditions [125].

Wang et al. (2020) established a HA-modified Mn–Zn ferrite magnetic nanoparticle-loaded block copolymer (MZF-HA) micellar system for cancer hyperthermia treatment (HT) that was used for synergistic therapy under alternating magnetic field (AMF) and radiation therapy (RT). MZF can generate thermal energy via AMF, leading to a local temperature increase of approximately 43 °C at tumor sites for mild HT, and the increased tumor oxygenation enhances the therapeutic effect of RT. In vitro experiments show that MZF-HA achieves high specific targeting of A549 cells, with excellent biocompatibility and improved performance under HT and RT conditions. The tumor volume decreased to 49.6% through the combination of HT and RT, compared to the 58.8% increase in the untreated group [126].

## Magnetic Nanoparticle-Based combination therapies incorporating immunotherapeutic approaches

Cancer immunotherapy is based on activating or enhancing the body’s immune response against cancer cells. Immunotherapy mechanisms include adoptive cell transfer, immune checkpoint inhibitor therapy, cytokine therapy, and cancer vaccines. These approaches aim to stimulate the immune system or overcome obstacles that prevent it from effectively recognizing and attacking cancer cells. Each approach targets one aspect of the immune response to improve its effectiveness against cancerous cells [127].

The history of cancer immunotherapy, from Coley’s approach to modern approaches such as checkpoint inhibitors and CAR T-cell therapy, demonstrates the significant impact immunotherapy has had on cancer treatment. Cancer immunotherapy uses the immune system to fight against cancer cells, which has led to substantial improvements in cancer treatment [128]. William Coley pioneered the use of bacterial toxins to stimulate the immune system against cancer cells [129]. In the mid-20th century, the application of cytokines such as interferons and interleukins in enhancing immune response was studied [130]. Research has shown that altering the immune response can reduce tumor size. Another approach is CAR T-cell therapy, which targets specific cancer antigens. Furthermore, cancer vaccines are designed to stimulate an immune response against specific or associated tumor antigens, and research is underway to improve their effectiveness [131].

Over the last decade, understanding the mechanisms of cells’ escape from the immune system has led to the successful immunotherapies, such as the use of immune checkpoint inhibitors and antigen-specific adoptive cell therapies [132].

There is a close cross-talk between metal ions and the immune system. Metal ions are widely involved in the cellular immune system, immune-related proteins, and immunotherapy. For example, in hyperthermia therapy, nanomedicine containing metal ions and thermal conversion not only effectively induces apoptosis in cancer cells, but also the released metal ions play a vital role in immune modulation [133].

The incorporation of nanoparticles into hyperthermia provides a non-invasive method capable of reaching deep-seated tumors within tissue [175]. Additionally, nanoparticles can reduce temperature heterogeneity within the cancer, thereby achieving an invariant response to a given thermal dose [134]. Intravenously injected nanoparticles of appropriate hydrodynamic size (10–100 nm) preferentially accumulate in tumors due to the enhanced permeability and retention (EPR) effect, which results from rapid tumor vasculature growth and a leaky vasculature with poor lymphatic drainage [135]. Additionally, the flexible surface chemistry and large surface-area-to-volume ratios of nanoparticles enable functionalization with targeting agents, such as peptides and antibodies, targeting overexpressed tumor receptors [136].

Therefore, nanoparticles can facilitate hyperthermia-based combination therapies by coadministration with therapeutics or by incorporating therapeutics directly into the nanoparticle design. These advantages provide the potential to enable novel combinational approaches, such as nanoparticle-mediated gene silencing of immune checkpoint ligands and mild NP-based hyperthermia, to boost a synergistic response [132].

Furthermore, it was suggested in some studies that iron ion overload can cause protumoral M2 to tumoricidal M1 tumor-associated macrophages repolarization, which has great significance in controlling the immunosuppressive tumor microenvironment [137]. For example, Rong et al. (2019) introduced Fe^3+^ ions to PEGylated polydopamine (PDA) to fabricate iron-chelated melanin-like nanocomposites, which could achieve exceptional Nanomaterial-based photothermal therapy and cause the release of tumor-associated antigens [138].

The production of ROS in tumor cells is a mechanism for killing cancerous cells exposed to radiotherapy. After cells are exposed to radiotherapy, water molecules are broken down, producing large amounts of ROS, including superoxide anions, hydroxyl radicals, and hydrogen peroxide [139]. These radiation-derived ROS can cause extensive oxidative damage to macromolecules, including DNA, lipids, and proteins, leading to a surpassing of the antioxidant capacity of tumor cells and activation of cancerous cell death pathways. ROS can activate the p53 signaling pathway, a pro-oxidant mediator, while suppressing antioxidant regulators such as SOD2 and Nrf2 [140, 141], and promote apoptosis through either death-receptor-mediated caspase-8 activation or mitochondrial injury leading to cytochrome c release. Beyond apoptosis, ROS-induced oxidative stress (OS) is essential for immunogenic cell death (ICD), characterized by the exposure and release of damage-associated molecular patterns (DAMPs), including CRT, HSPs, HMGB1, and ATP [142]. Excess ROS triggers ER stress via PERK and IRE1α phosphorylation, promoting CRT surface exposure as a signal for dendritic cells [143–147]. Mitochondrial ROS can further support ICD by activating the RIP1-RIP3 necrosome pathway [148, 149] and generating oxidized mitochondrial DNA that triggers the STING-TBK1-IRF3-IFN-β axis in dendritic cells, thereby enhancing CD8⁺ T-cell priming [150]. ROS also induce lysosomal membrane permeability, resulting in the release of cathepsin D and caspase-8, which can further activate Bid and amplify CRT exposure [151, 152]. In addition, ROS can modulate broader antitumor immune responses; this means moderate levels enhance phagocytosis, antigen presentation, and T-cell activation, whereas excessive ROS activate T-cell survival and function [153].

## Targeted and triggered drug delivery using magnetic nanoparticles and physical methods

Advanced drug delivery methods aim to reduce drug dosage and side effects while increasing treatment efficacy. Magnetic fields have minimal impact on tissues and have been explored to direct nanoparticles for targeted drug accumulation and release. This reduces drug exposure to non-target tissues, enhancing therapeutic efficiency [154, 155].

The proposed drug delivery mechanisms must keep the therapeutic drug dose concentrations at targeted tissues while minimizing off-target effects [156]. Regarding this concept, several nanocarriers have been developed to improve tumor-specific drug accumulation and controlled release process, including polymer-drug conjugates [96], dendrimers [157], nanogels [158], metallic nanoparticles [159], mesoporous silica nanoparticles [160], virus-like particles [161], lipid nanoparticles [162], and polymer nanoparticles [163].

Nanocarrier can be directed at the target site via direct injection [164], passive targeting (enhanced permeability and retention) [165], or active targeting mechanisms such as ligand targeting [166], acid-dependent targeting [167], matrix metalloproteases [168], radiotherapy-induced targeting [169], and magnetic targeting [170]. The releasing process from these carriers occurs through leakage, enzymatic degradation, or external stimuli like acidity, hypoxia, ultrasound, light, or temperature [171, 172].

**Fig. 1:**
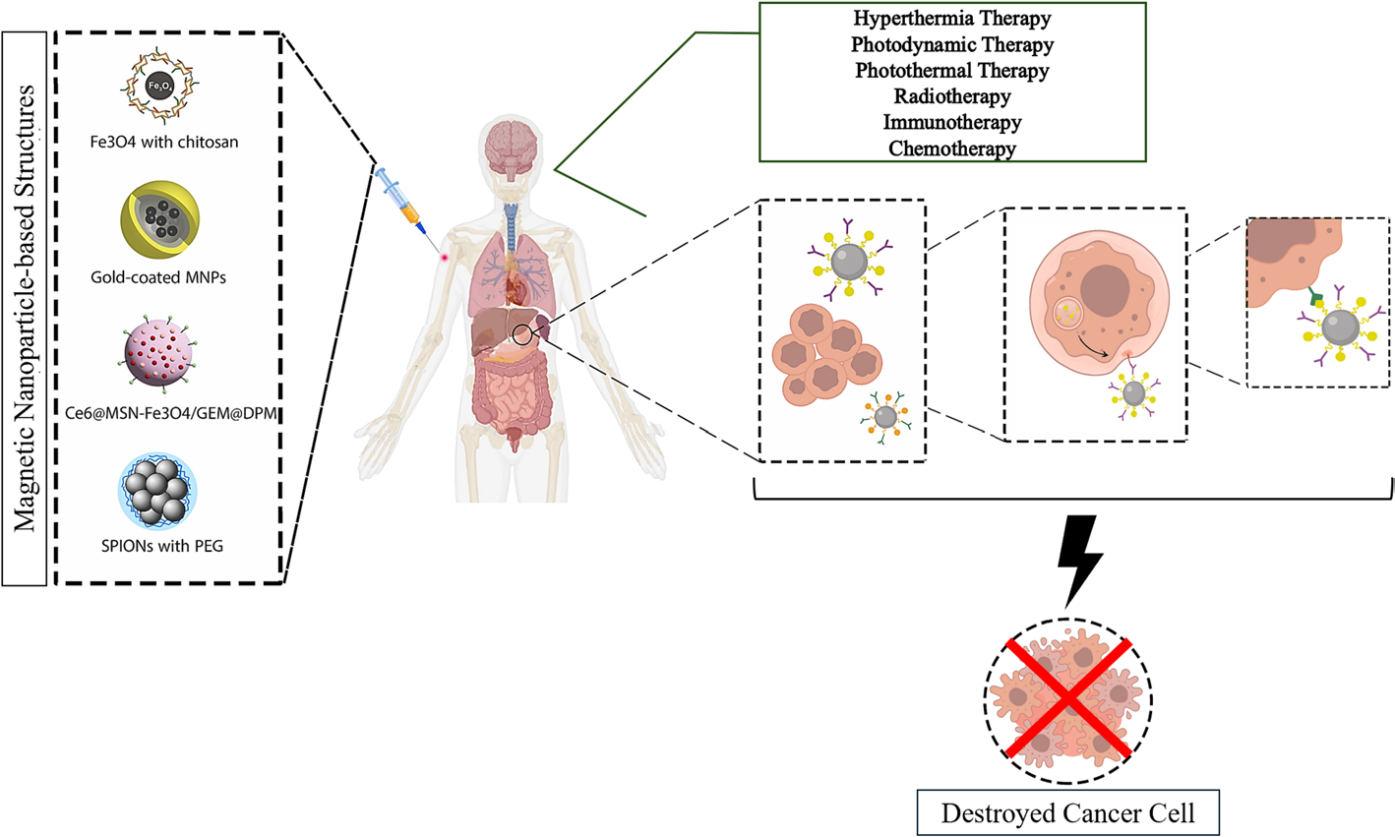
Cancer treatment using MNP in combination therapy. MNP can be applied in drug delivery systems for cancer combination therapies to enhance the therapeutic efficacy

However, strategies relying on molecular and environmental cues may face limitations due to tissue heterogeneity and non-selectivity [173, 174]. Furthermore, surrounding tissues may absorb exogenous energy sources such as light or ultrasound. Bio-orthogonal magnetic drug release is a promising approach in which magnetic fields penetrate tissues without significant absorption or directional changes. Magnetic drug targeting strategies include magnetic particles to enhance drug accumulation via magnetic guidance [175, 176] and heating magnetic particles to induce localized hyperthermia at the target site [177]. Additionally, magnetic fields can activate drug release from temperature-sensitive carriers [178, 179]. Figure 1 shows a schematic of MNP use in a drug delivery system in combination with other cancer treatments.

These approaches demonstrate the potential of magnetic nanoparticles as versatile tools for cancer therapy, offering targeted drug delivery, hyperthermia, and radiosensitisation to improve treatment outcomes (Table 2).

**Table 2:** Targeted and triggered drug delivery using magnetic nanoparticles and physical methods has been reported in different studies

Drug Name	Cancer Type	Physical Trigger Method	Nanoparticle Type	Size / Hydro Dynamic diameter	Delivery Mechanism	Surface Coating	Key findings	Ref
Doxorubicin	Breast Cancer	Magnetic Field	SPIONs with PEG	60–120 nm	Magnetically guided, heat-sensitive release	Covalently attached PEG layers with folic acid ligand on the outer surface for active targeting.	Enhanced uptake in MCF-7 cells overexpressing folate receptors; improved colloidal stability, suitable for targeted DOX delivery and theragnostic	[180]
Paclitaxel	Fibrosarcoma	Hyperthermia Therapy	Fe3O4 with chitosan	100–200 nm	Heat-induced drug release	Cs PTX PEG FA coating (chitosan containing PTX, grafted with PEG and folic acid).	Stable, nanoscale SPION@Cs PTX PEG FA; inhibited proliferation and induced apoptosis in fibrosarcoma cells; reduced tumor volume and increased survival in mice.	[181]
Cisplatin	Lung Cancer	Magnetic Field + pH	Fe3O4 with pH-sensitive coating	100–200 nm	Eliminate the limitations of cisplatin	Biodegradable triblock copolymer PCL PEG PCL shell.	Drug-free nanoparticles were biocompatible; cisplatin loading decreased IC50 and enhanced antiproliferative activity in A549 cells	[182]
Curcumin	Sarcoma	Hyperthermia + Magnetic Field	Fe3O4@PLA-PEG/curcumin	Nanoparticle core 8.5 nm Hydrodynamic nanometer 10–100 nm	Drug delivery and release of nanocarriers	Diblock copolymer PLA PEG shell	Magnetically controlled sustained release; effective MRI contrast; strong tumor regression with magnetic hyperthermia in mice	[183]
Gemcitabine	Pancreatic Cancer	Photodynamic Therapy	Ce6@MSN-Fe3O4/GEM@DPM	100–200 nm	Drug delivery and release of nanocarriers	Functionalized mesoporous silica surface	Enabled co-delivery of gemcitabine and PS; improved killing of pancreatic cancer cells vs. gemcitabine alone	[184]
5-Fluorouracil	Tumor cells	Hyperthermia Therapy	Fe3O4/PNIPAM/5-Fu@mSiO2–CHI/R6G	10–100 nm	Magnetic target therapy	PNIPAM inner shell + mSiO2 shell + outer chitosan layer with R6G.	Temperature-dependent release; high drug loading; strong tumor cell uptake; useful for chemo, hyperthermia, and imaging.	[185]
Camptothecin	Pancreatic Cancer	Alternating Magnetic Field (AMF)	Fe3O4@MSNs	100–200 nm	Drug delivery and release of nanocarriers	Mesoporous SiO2 shell (inorganic)	Efficient drug loading; heating accelerated release; PTX/CPT Fe3O4@MSN increased cytotoxicity in PANC 1 cells	[186]
Temozolomide	Glioblastoma	Radiotherapy	SPION@PEG-PBA-PEG nanoparticles (TMZ-MNP-FA NPs)	100–200 nm	Magnetic hyperthermia	PEG PBA PEG shell with folic acid	Acted as T2 MRI agent; enhanced uptake in C6 glioma cells; combined AMF + chemo + radiotherapy produced the highest anticancer efficacy	[187]

## Accumulation of nanoparticles and penetration into tumors by magnetic field

Nanoparticles accumulate poorly at the tumor site. Wilhelm et al. confirmed that nanoparticle accumulation at the tumor site occurred via passive targeting, with a low concentration of 0.6% of the total dose. However, the active target increases nanoparticle accumulation by only 0.3%. The researchers believe that the low accumulation of nanoparticles is due to the high hydrostatic pressure in tumor tissue [188]. On the other hand, the tumor’s heterogeneity poses challenges for drug delivery to the tumor site. Nanocarriers accumulated using the passive targeting method barely penetrate the interstitium. The drug may not affect areas far from blood vessels, since interstitial hydrostatic pressure and the extracellular matrix are involved in the transport of drug transporters outside the vessels [189, 190]. Therefore, methods that increase the penetration of drug transporters and their accumulation in the tumor site seem necessary.

Magnetic targeting has been proposed as an efficient mechanism to enhance the accumulation and penetration of magnetic drug transporters into tumors [191]. The drug and the magnetic nanoparticle are combined into a magnetic target, and an external constant magnetic field is used to accumulate the drug carrier in the target tissue near the magnet. For example, Marie and others used a magnet to create a constant magnetic field. Increase the accumulation of magnetoliposomes at the site of glioblastoma tumors in mice [192]. Huang et al. (2012) studied and tested doxorubicin loaded in magnetic micelles in a squamous cell carcinoma model [193].

Magnetic targeting has been confirmed in humans. As early as 1996, Lübbe et al. confirmed that magnetic drug targeting could increase the concentration of epirubicin-conjugated nanoparticles in sarcomas in phase 1 and 2 clinical trials [194]. Despite the advantages of using a magnetic field to target magnetic nanoparticles, this area also has limitations. The first problem is the loss of magnetic field strength with distance, limiting its influence to short distances. Therefore, the magnetic field can only target nanoparticles to superficial tumors. In studies by Lübbe et al. (2001), the target of magnetic preparations was limited to tumors within 5 mm of the body surface [194]. Rotariu et al. (2005) also calculated that using particles smaller than 500 nm with typical magnetic properties, combined with fields generated by earth element magnets, could only affect tumors within 18 mm of the body surface. For large particles of about 5 micrometers, depths of up to 15 cm can also be targeted [195]. However, intravenous injection of particles below 200 nm increases their residence time in the bloodstream [196]. So, using these particles to target deeper tumors is not practical.

**Fig. 2:**
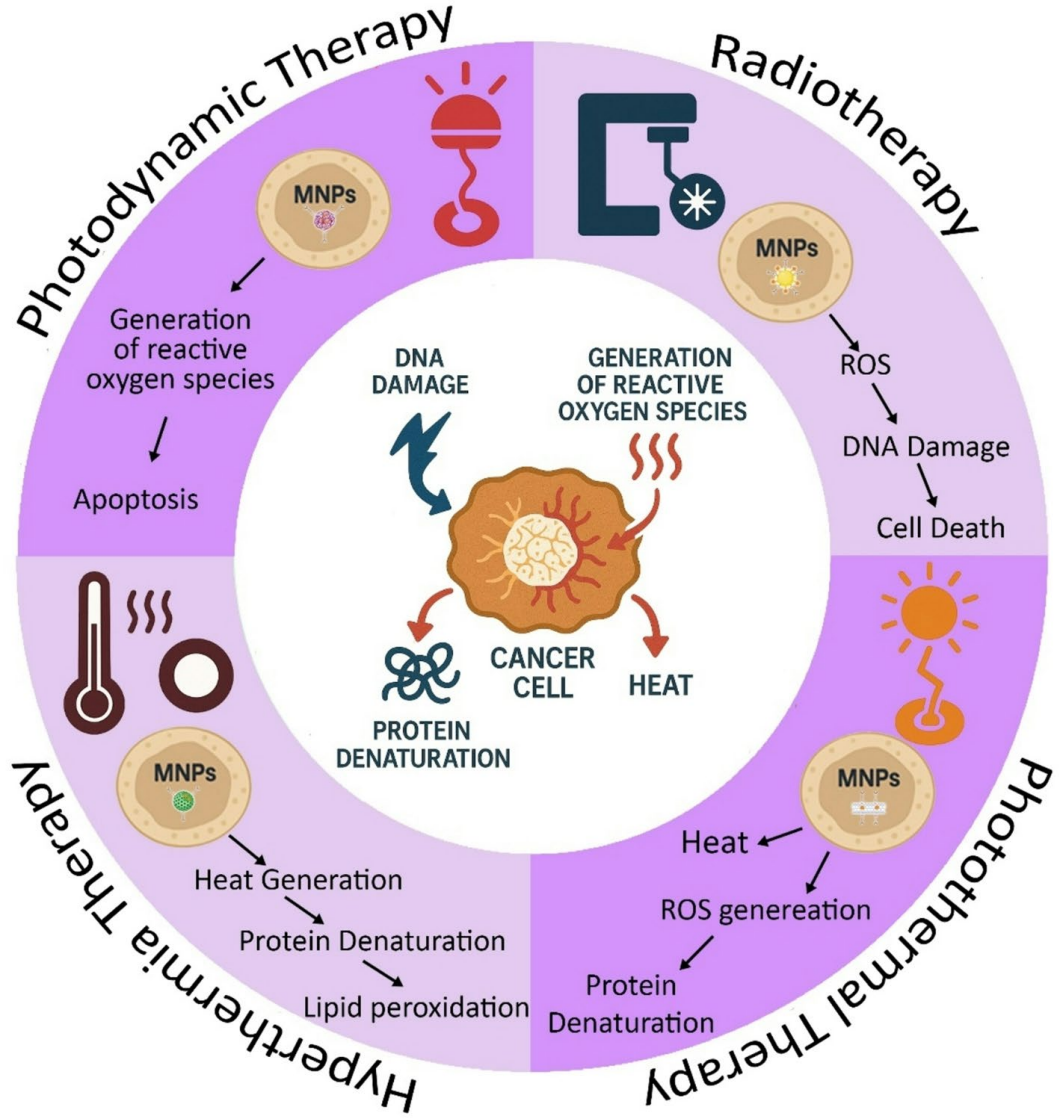
The efficacy of Magnetic Nanoparticles in the Synergic Mechanism of Hyperthermia, Photothermal therapy, Photodynamic therapy, and Radiotherapy. Magnetic nanoparticles can act as mediators and enhancers of therapeutic methods in combined systems for cancer treatment

These findings confirm the multifunctionality of MNPs as radiation sensitizers, heat generators, ROS producers, and protein denaturants, which increases their appeal in cancer research (Fig. 2).

## Translational challenges and limitations of magnetic nanoparticles in combination cancer therapy

Several studies on magnetic nanoparticles confirm that their toxicity is due to their reactivity and to oxidative stress related to iron [197]. In vitro studies show that uncoated or unstable iron oxide nanoparticles induce ROS production. Surface coatings for nanoparticles, such as silica-dextran or PEG, reduce the effects described above. These coatings provide colloidal stability and prevent direct interaction of the nanoparticles with the cell membrane [198, 199]. However, the risk of generating ROS molecules remains high [197]. The main limitation of the therapeutic use of magnetic nanoparticles is their heterogeneity and unpredictable distribution in the body after injection. Furthermore, another limitation is achieving the necessary magnetic nanoparticle penetration depth during hyperthermia to generate the heat required to destroy tumor cells. Thus, when the tumor is located deep within the body, the risk of tissue damage, particularly to nerves, induced by the temperature rise during hyperthermia is increased [200].

The long-term retention of magnetic nanoparticles in the body is another challenge in working with them. Since iron oxide cores are often degraded slowly in lysosomes, this poses problems for in vivo use [197]. In other words, as reported in the articles, many magnetic nanoparticles are coated with various polymers or silica, which improves their colloidal stability, drug loading, and tumor targeting. However, in vivo, these coatings degrade in acidic environments, thus compromising drug delivery and tumor targeting [201].

Animal studies have confirmed that even tiny paramagnetic iron oxide nanoparticles persist in the liver and spleen for several months, making it challenging to use repeated doses of nanoparticles in procedures such as hyperthermia or MRI, and raising concerns about inflammatory responses [202].

Another problem associated with the use of iron oxide nanoparticles is their uptake by the RES, particularly hepatocytes. Studies in rodents have shown that iron oxide nanoparticles, injected intravenously, accumulate in the liver and spleen within minutes to hours, thereby shortening the time these nanoparticles spend in the circulation and, as a result, delivering lower levels to tumor tissue [203]. High iron levels in the body resulting from magnetic iron nanoparticles are another risk, especially with repeated, high-dose use. Studies on mouse liver cell lines and macrophage cells show that high iron concentrations resulting from these.

nanoparticles can catalyze Fenton reactions and also increase lipid peroxidation [204]. Changes in iron concentrations in the body can be a risk factor for patients with iron metabolism disorders [205].

Translational challenges with magnetic iron nanoparticles are primarily due to nanoparticle coating instability and their toxicity. However, until now, even well-functioning nanoparticle formulations have not proposed perfect and acceptable results in the clinical phase [206]. Recent advances in MNPs have shown that, although nanotherapies may offer a range of solutions to unsolved problems, only a small number of these findings reach the world market. For this reason, establishing good manufacturing practice (GMP)-compliant nanoparticle manufacturing at a large scale is necessary for a successful transition to clinical trials. Enhancing particle quality, such as size and distribution, according to pharmaceutical standards, is an important aspect, in addition to the choice of manufacturing methods [207]. Therefore, researchers need to determine the precise characteristics of these nanoparticles and the degree of GMP and immune response [208].

## Conclusion

Magnetic nanoparticles can be used in many therapeutic methods due to their special physical structure and magnetic properties. Their paramagnetic properties make them suitable candidates for drug delivery via a magnetic field. These features include biocompatibility, large surface area, and magnetic torque, which can be adjusted during manufacturing for specific applications [72].

Iron oxide nanoparticles can be controlled within the body by an external magnetic field, making them suitable for combined use. These nanoparticles can absorb laser light, generate heat via photothermal effects, and, in radiation therapy, cause more significant damage to cancer cells by sensitizing DNA [74, 76, 209]. Also, applying a magnetic field increases the temperature in tumor tissues containing magnetic nanoparticles, thereby killing cancer cells. The paramagnetic property helps them be directed to the tumor site by the magnetic fields within the body. Magnetic nanoparticles become paramagnetic in a magnetic field, making them suitable candidates for magnetic targeting. Given the ability of cancer cells to adapt to treatments, including chemotherapy, radiation therapy, and hyperthermia, the best method is the simultaneous use of modalities on cancer cells. The mechanism of cancer cell adaptation involves non-superficial tumor cells that receive a dose below the therapeutic level. The combined treatment methods increase the likelihood of cancer cell death in deeper parts of the tumor at doses below the therapeutic dose. This is achieved by altering the membrane of cancer cells, thereby increasing their permeability to anticancer drugs. On the other hand, many tumors exhibit heterogeneity. That is, different cell populations in the tumor can respond differently to each therapeutic method. Hence, combining therapeutic methods increases the likelihood of eliminating even heterogeneous tumor populations. In addition, combining methods allows lower doses, for example, in radiotherapy or chemotherapy. However, despite the potential of MNP in cancer treatment, which has generated considerable interest in cancer research, ongoing investigations to explore their capabilities and refine their applications are being conducted. In this regard, several obstacles must be overcome to guarantee the translation of laboratory experimentation to practical application. These issues include technical perspectives, such as large-scale and GMP-compliant production, process control, and batch reproducibility; economic issues, as well as overall cost-effectiveness; and biological issues, including biodistribution investigations, biocompatibility, biodegradation, and biosafety.

## Data Availability

No datasets were generated or analysed during the current study.
